# Spatial evolution of human cultures inferred through Bayesian phylogenetic analysis

**DOI:** 10.1098/rsif.2022.0543

**Published:** 2023-01-04

**Authors:** Takuya Takahashi, Yasuo Ihara

**Affiliations:** ^1^ Meiji Institute for Advanced Study of Mathematical Sciences (MIMS), Meiji University, Nakano 4-21-1, Nakanoku, Tokyo 164-8525, Japan; ^2^ Department of Biological Sciences, the University of Tokyo, Hongo 7-3-1, Bunkyoku, Tokyo 113-0033, Japan

**Keywords:** cultural evolution, spatial dynamics, Bayesian phylogenetic analysis, coalescent theory, network, population genetics

## Abstract

Spatial distribution of human culture reflects both descent from the common ancestor and horizontal transmission among neighbouring populations. To analyse empirically documented geographical variations in cultural repertoire, we will describe a framework for Bayesian statistics in a spatially explicit model. To consider both horizontal transmission and mutation of the cultural trait in question, our method employs a network model in which populations are represented by nodes. Using algorithms borrowed from Bayesian phylogenetic analysis, we will perform a Markov chain Monte Carlo (MCMC) method to compute the posterior distributions of parameters, such as the rate of horizontal transmission and the mutation rates among trait variants, as well as the identity of trait variants in unobserved populations. Besides the inference of model parameters, our method enables the reconstruction of the genealogical tree of the focal trait, provided that the mutation rate is sufficiently small. We will also describe a heuristic algorithm to reduce the dimension of the parameter space explored in the MCMC method, where we simulate the coalescent process in the network of populations. Numerical examples show that our algorithms compute the posterior distribution of model parameters within a practical computation time, although the posterior distribution tends to be broad if we use uninformative priors.

## 1. Introduction

The human is characterized by the abundance of cultural traits, such as toolkits, potteries, languages, religions and modern skills, the evolution of which has long been analysed in parallel with genetic traits [1,2]. While genetic traits are inherited from one generation to the next through reproduction, cultural traits are transmitted from individual to another via social learning, such as imitation, emulation and teaching. Unlike the evolution of genetic traits, which are transmitted exclusively from parents to offspring, except the case of horizontal gene transfer, cultural evolution is somewhat more complicated due to the existence of horizontal transmission, whereby a cultural trait is transmitted between individuals in the same generation [3].

Horizontal transmission is one of the factors underlying the emergence of spatial or geographical patterns of many cultural traits, adding a layer of complexity to the question of how these patterns have formed; spatial distribution that we observe today can be explained by the past divergence of populations and spatial interaction between populations [4]. Regarding the former, when a small group of people splits from a large population and migrates to an uninhabited area to form a new population, the cultural traits of the new and parent populations become gradually different as they accumulate mutations independently (i.e. cultural macroevolution [3]). Analogous to the case of biological speciation, therefore, the cultural distance (i.e. dissimilarity) between two populations is expected to be positively correlated with the genetic distance, or the time elapsed since the population divergence, as has been observed in the variations of lexicon [5] and folk songs [6]. As for the latter, transmission of cultural traits between individuals plays a pivotal role in the formation of spatial patterns in cultural traits. Since human contact is more intense between geographically closer individuals, traits are likely to diffuse gradually from nearby populations to farther areas. The interplay of these two factors complicates the spatial evolution of cultural traits [4,7].

Transmission of cultural traits or social information in space has been modelled by means of networks [8–11], where the nodes represent either individuals or populations. Although the network is a powerful tool to represent the cultural transmission among populations, these previous models are mostly theoretical and hardly applicable to empirical data to make inference. Concerning statistical inference with a network, another previous study applied a network of speakers to the geographical distribution of English folk speech observed in a linguistic atlas and inferred the copying process of each survey item by a maximum-likelihood analysis [12]. However, this model did not quantify mutation events, whereby a trait variant is replaced by another, which is also an important underlying factor of the spatial evolution of cultural traits.

The current study aims to develop a new statistical model which enables the inference of the cultural dynamics among multiple populations based on observed data. We focus on a cultural trait which takes one out of multiple possible states or variants, such as the presence/absence of a given belief, folk song, etc. or different types of pottery patterns. Suppose that we have data that specify the state of the focal cultural trait (hereafter ‘cultural states’) in multiple populations, typically given as a map on which cultural states are plotted for multiple locations that are culturally interdependent. We will estimate the frequency of cultural transmission among populations, the mutation rate between different states, and the states of populations which are yet to be observed. To achieve this goal, we will establish a Bayesian framework under a coalescent model in a population network, which requires us to combine expertise of two different techniques: (i) coalescent theory on a population network and (ii) Bayesian phylogenetic analysis. Here we will explore the previous studies of these two fields one by one.

First, coalescent theory is a retrospective approach to population genetics originally developed in order to model the amount of genetic polymorphism in a population of a single species [13,14]. A coalescent process starts from multiple copies of a gene in question sampled in the present generation, tracing the lineage of these copies by simulating their parents every generation in the past, which yields the genealogical tree of all the sampled genes. To treat the spatial structure of populations, some coalescent models consider a metapopulation, which constitutes a group of multiple populations, and integrate the effect of migration between populations [15,16]. Models of coalescent process have gradually been adapted to cultural traits, and recent theoretical studies applied the coalescent process to the transmission of cultural traits [17,18]. We have previously developed a retrospective model of trait evolution in a network of populations [11], which is thus far the most relevant to the current study. The model we will describe in the current article is an extreme case of the structured coalescent with population size 1 for each subpopulation, the transmission among which is represented by a weighted network.

The other method that we borrow in this article is the Bayesian approach for phylogenetic reconstruction, which has been a powerful method in molecular phylogenetics. In addition to the well-known methods to reconstruct a phylogenetic tree from molecular data, a large body of literature has described the Bayesian framework to estimate the mutation rates and the ancestral states of morphological traits based on a given phylogenetic tree [19,20]. Beyond the genetic data, phylogenetic approaches have often been applied to analysing cultural traits such as political complexity [21] and folk tales [22]. Besides, linguists pay much attention to the Bayesian approach to reconstruct the linguistic phylogeny [23], estimate the replacement rates of vocabulary [24,25] and infer the originating place of a language family [26].

Here we will introduce a Bayesian framework based on both the coalescent process in a network and trait evolution on a single-trait tree, which will work as follows. First, we will model the spatial structure of populations by a network where the nodes represent populations and the edges are weighted by the transmission rate of the cultural trait of interest. Second, following the ancestral process in a network described in Takahashi and Ihara [11], we will compute the prior probability of the history of cultural transmission over the past generations. Third, we trace the coalescent process of trait copies observed in the present generation, to obtain the genealogical tree whose taxa represent trait variants observed in different populations. Fourth, based on the observed states of the cultural trait, we compute the likelihood of the tree. We will perform a Markov chain Monte Carlo (MCMC) method to estimate the mutation rates between trait variants or states, the transmission rates among populations, the genealogical relationship among the present trait copies, and the states of unobserved populations.

Note that the tree in our model represents the genealogy of a single cultural trait rather than the phylogeny underlying the evolution of multiple traits. Hence, if we have multiple traits of interest, we should apply the model independently to each of them. As each trait can independently diffuse in the network, this model setting allows us to represent the horizontal transmission of the trait by tracing the past locations of trait lineages. In this sense, our approach may be similar to that of the multi-species coalescent model [27] which treats multiple gene trees embedded in the species tree, or that of the model of lexical borrowing which treats multiple word trees that are distinct from the language tree [28].

This paper is structured as follows: in the ‘Theory’ section, we will describe the assumptions of the probabilistic model and the algorithm for Bayesian inference based on the model. Since this algorithm is computationally expensive, we will also introduce a heuristic algorithm to approximate the posterior distribution of model parameters and genealogical trees. In the ‘Example on a square grid’ section, we will apply both the exact algorithm and heuristic approach to a simplistic case where populations are aligned like a two-dimensional square grid, aiming at investigating to what extent our method is practical.

## 2. Theory

### 2.1. Basic model and observed data

We consider a network of *n* populations denoted by *P*_1_, … ,*P_n_*, which mutually transmit their cultural traits. The spatial structure in our model is thus discrete, representing for instance distinct human groups like cities, villages or islands.

**Figure 1.**
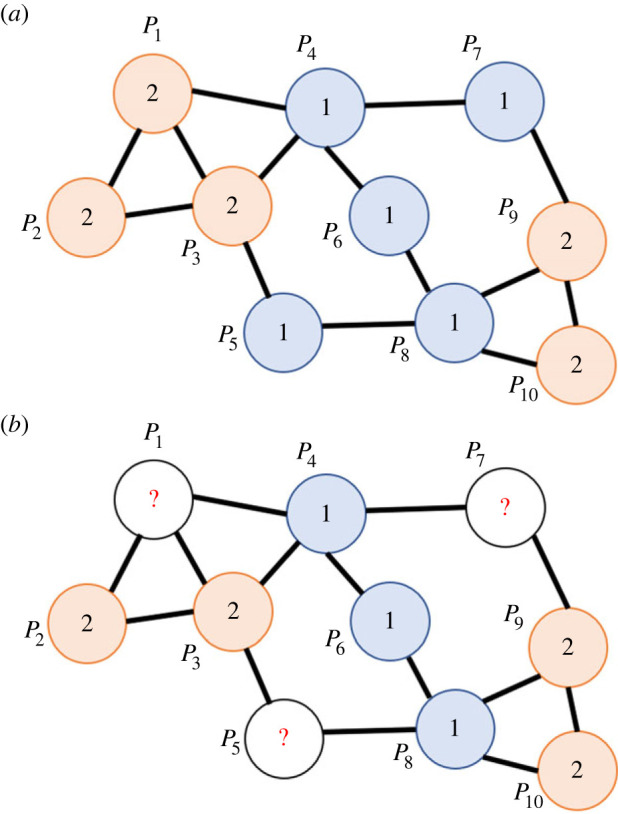
Network of populations assigned with cultural states. Circles: populations; edges: presence of cultural transmission; blue: state 1; orange: state 2; white circle with a question mark: unknown state. (*a*) Network with 10 populations with two possible cultural states (i.e. *n* = 10, *k* = 2). (*b*) Example of an observed dataset. Cultural states of 7 out of 10 populations in the network of panel (*a*) are observed.

Every population (node) in the network takes one out of *k* possible cultural states, denoted by positive integers 1, … ,*k* (figure 1*a*). For instance, *k* = 2 represents a binary state, such as the presence/absence of a focal cultural trait like a ritual, belief or custom, while *k* ≥ 3 indicates a more diversified trait, such as different patterns of potteries and toolkits, as well as dialect words for the same object. The state taken by *P_i_* is denoted by *S*(*i*). Note that, unlike standard subpopulation models used in the field of population genetics and cultural evolution, the state is assigned to each population, instead of each individual, meaning that our model does not consider the diversity of the focal trait within a single population.

We assume that cultural states of *m* populations $P_{y_{1}},\cdots ,P_{y_{m}}$ have already been observed, where 1 ≤ *m* ≤ *n*, 1 ≤ *y_i_* ≤ *n* (1 ≤ *i* ≤ *m*) and *y_i_* ≠ *y_j_* (*i* ≠ *j*) hold true. Thus, we have observed data ***Y*** = { *S*(*y*_1_), … ,*S*(*y_m_*)}. In other words, the states taken by some of the nodes are known, whereas the others are not. An example of a dataset is given in figure 1*b*, in which cultural states in 7 out of 10 populations in the network of figure 1*a* are known. We will use this kind of dataset to infer the transmission rates among populations, mutation (transition) rates among states and genealogical relationship among the observed copies of the cultural state. One of the most suitable datasets for this model is map data, in which variants of the cultural trait in question are plotted at multiple locations.

### 2.2. Dynamics of the probabilistic model

**Figure 2.**
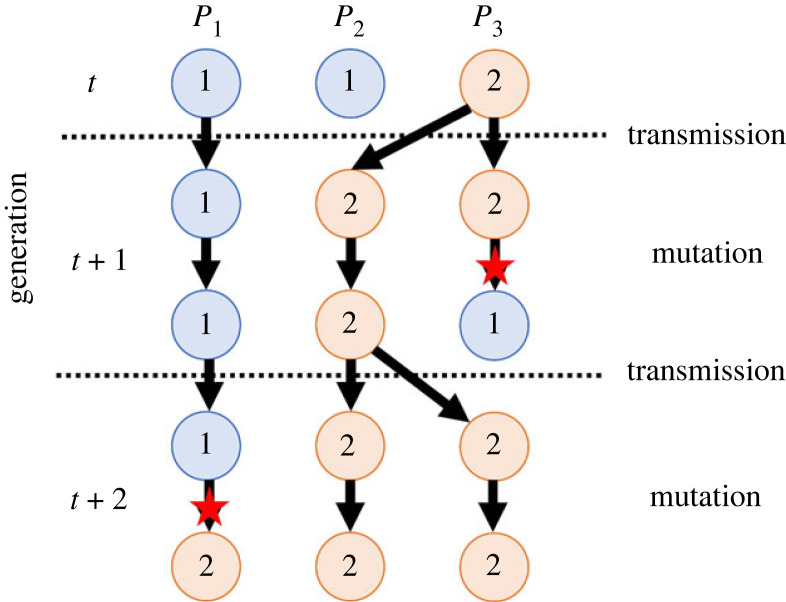
Dynamics of cultural states of three populations in three consecutive generations (*n* = 3, *k* = 2). Circles: populations; blue: state 1; orange: state 2; arrows: inheritance of cultural states; red stars: mutation events.

To develop a probabilistic model for the Bayesian inference, we define how the cultural state at each node of the network evolves over time. We assume a time-discrete model where each time step sees two events: transmission and mutation (figure 2). Note that, as we mainly focus on the case where the time unit is considered as the length of a human generation, we will interchangeably employ the two terms ‘time step’ and ‘generation’ in this paper.

At the beginning of each time step, every population chooses one population, including itself, and learns (copies) its cultural state taken at the previous time step (i.e. transmission event). The probability that *P_i_* copies the state of *P_j_* is given by *a_ij_*, which we refer to as the transmission rate from *P_j_* to *P_i_*. Thus, the edges of the population network are seen to be weighted by the transmission rates, where the matrix

$$A=\left(\begin{array}{ccc} a_{11} & \cdots & a_{1n}\\ \vdots & \ddots & \vdots \\ a_{n1} & \cdots & a_{nn} \end{array}\right)\;$$

is regarded as the adjacency matrix of the graph. Since *a_ij_* is stochastic, its summation over *j* gives one for every *i*.

After copying a cultural state from the previous time step, the inherited state may change into another through a mutation event, whereby the state *i* mutates into *j* with probability *q_ij_*, which we call the mutation rate from *i* to *j*. For notational simplicity, we define *q_ii_* as the probability that the state *i* stays unchanged during the mutation event. The mutation event can thus be seen as a Markov process, where the probability transition matrix is given by

$$Q=\left(\begin{array}{ccc} q_{11} & \cdots & q_{1k}\\ \vdots & \ddots & \vdots \\ q_{k1} & \cdots & q_{kk} \end{array}\right).$$

Since ***Q*** is probabilistic, every row sums up to one.

### 2.3. Parameter reduction

Although we are interested in estimating the transmission rates *a_ij_*, we would need to estimate *n*(*n* − 1) independent parameters without imposing any constraint on the transmission rates, which would be unrealistic when we consider a network with many nodes. To reduce the number of parameters, we assume *a_ij_* is given as a function of *i*, *j*, and a set of parameters ***θ_τ_*** which we refer to as the ‘transmission parameters':

$$a_{ij}=f_{\tau }(i,j,\theta_{\tau }).$$

For instance, if the network is considered as a complete graph (island model), transmission can be modelled by assuming that the rate of inter-population transmission is constant as follows:

2.1a $$a_{ij}=\{\begin{array}{c} 1-c\;(i=j)\\ \frac{c}{n-1}\;(i\neq j) \end{array},\;$$

in which case ***θ_τ_*** = {*c*}. If we hope to model the difference in the rate at which populations learn from a different population, we might use a model like

2.1b $$\;a_{ij}=\{\begin{array}{c} 1-c_{i}\;(i=j)\\ \frac{c_{i}}{n-1}\;(i\neq j) \end{array}.\;$$

In this case, the set of transmission parameters would be written as ***θ_τ_*** = {*c*_1_, … ,*c_n_*}, each following a single prior distribution *P*(*c*).

If geographical and demographic information is available, the following model may be appropriate (cf. Burridge [29]):

2.1c $$a_{ij}=\frac{\pi_{j}\exp(-(d_{ij}^{2}/2\sigma^{2}))}{\sum_{{\;j}^{\prime }=1}^{n}\pi_{{\;j}^{\prime }}\exp(-(d_{i{\;j}^{\prime }}^{2}/2\sigma^{2}))},$$

where *π_j_* denotes the population size of *P_j_*, and *d_ij_* represents the geographical distance between *P_i_* and *P_j_*. As the denominator of equation (2.1*c*) is a normalizing factor, this equation assumes that populations are likely to learn from large populations and nearby populations. In this case, ***θ_τ_*** = {*σ*}.

Similarly, to reduce the parameters concerning mutation rates *q_ij_*, we assume

$$q_{ij}=f_{\mu }(i,j,\theta_{\mu }),$$

where ***θ_μ_*** is a set of parameters which we refer to as the ‘mutation parameters'. If the mutation rate is considered common to every pair of two states, we have

2.2 $$q_{ij}=\{\begin{array}{c} 1-\mu \;(i=j)\\ \frac{\mu }{n-1}\;(i\neq j) \end{array},$$

in which case the set of mutation parameters is given by ***θ_μ_*** = {*μ*}.

Instead of directly inferring transmission rates *a_ij_* among populations and mutation rates *q_ij_* among states, the current Bayesian framework will infer the transmission and mutation parameters (i.e. ***θ_τ_*** and ***θ_μ_***).

### 2.4. History of cultural transmission

**Figure 3.**
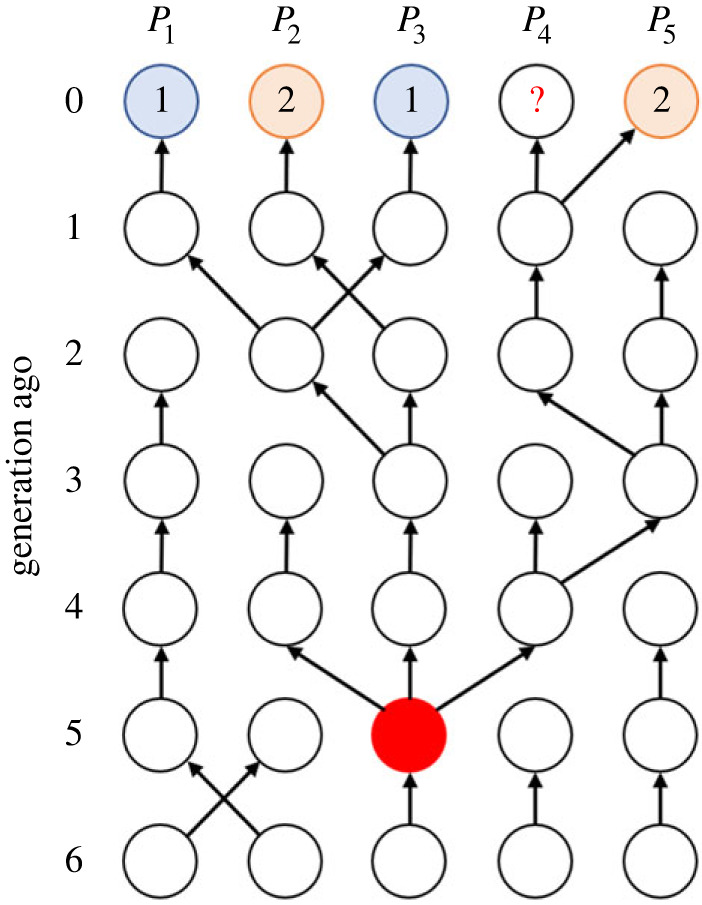
History of cultural transmission of all *n* (= 5) populations during the past six generations. Circles represent populations and arrows represent cultural transmission from the immediately previous generation. Note that states in four out of the present populations are included in the observed dataset. Red circle represents the most recent common ancestor of the four known state copies, which appears in *P*_3_ five generations ago.

To perform Bayesian inference, we need to calculate the probability (likelihood) that the observed set of cultural states are realized. Since the cultural states have been transmitted from generation to generation, the current states are probabilistically dependent on the history of transmission, which is depicted in figure 3.

To encode the information about the past transmission, we first need to consider the age of the most recent common ancestor (MRCA), which is defined as the youngest common ancestor that every observed copy of the state shares. In the case of figure 3, we have four observed copies of cultural states in the present generation (*m* = 4). As all four states included in the dataset are descendants of the state copy that was taken by *P*_3_ five generations ago, the time to the most recent common ancestor (TMRCA) is calculated as five generations in this case.

Here, our model requires the prior knowledge of the maximum possible value of the TMRCA, which is denoted by *τ*. We consider a matrix ***G*** of dimension *τ* × *n*, whose element at the *i*-th row and *j*-th column represents the population (i.e. subscript) from which *P_j_* copied the state *i* − 1 generations ago.

For example, if we choose *τ* = 6, the history of the transmission depicted in figure 3 is written as:

2.3 $$G=\left(\begin{array}{ccccc} 1 & 2 & 3 & 4 & 4\\ 2 & 3 & 2 & 4 & 5\\ 1 & 3 & 3 & 5 & 5\\ 1 & 2 & 3 & 4 & 4\\ 1 & 3 & 3 & 3 & 5\\ 2 & 1 & 3 & 4 & 5 \end{array}\right).$$

Note that ***G*** only contains the information about the transmission events during the past *τ* generations, but as it is known that the MRCA is *τ* generations or younger, ***G*** contains the information of how the present state copies have split and descended from the common ancestor. Hence, letting *S* denote the cultural state of the MRCA, observed states in the present generation are probabilistically dependent on ***G***, *S* and the mutation parameters ***θ_μ_***.

### 2.5. Probabilistic dependency among variables and Bayesian inference by Markov chain Monte Carlo method

**Figure 4.**
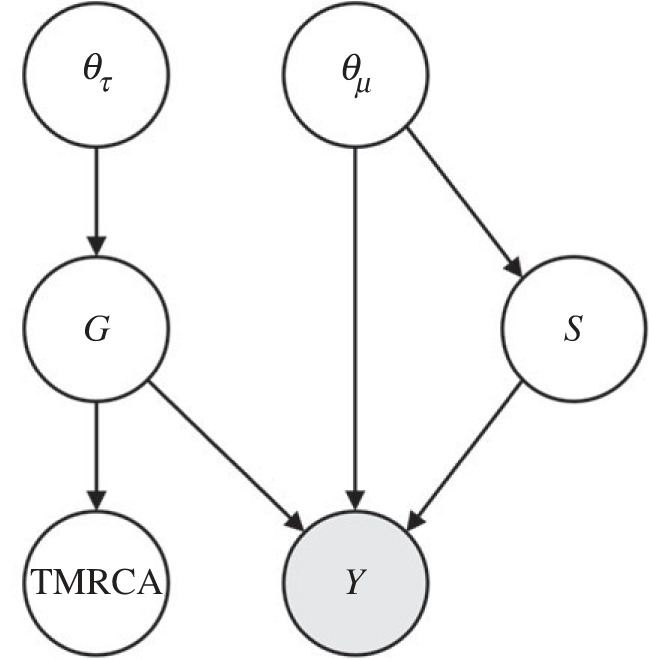
Graphical representation of the probabilistic dependency of variables. Grey circle represents observed variables.

Figure 4 represents the probabilistic dependency of model variables. Define ***D*** as

D≡Y∩{TMRCA≤τ}.

The joint posterior distribution is obtained by Bayes' rule as follows:

2.4 $$\begin{array}{cc} P(\theta_{\tau },\theta_{\mu },G,S|D)\; & =\frac{P(D|\theta_{\tau },\theta_{\mu },G,S)P(\theta_{\tau },\theta_{\mu },G,S)}{P(D)}\;\;\\ \; & =\frac{P(D|\theta_{\mu },G,S)P(S|\theta_{\mu })P(G|\theta_{\tau })P(\theta_{\tau })P(\theta_{\mu })}{P(D)}.\; \end{array}$$

Neglecting the marginal likelihood *P*(***D***), we have

2.5 $$P(\theta_{\tau },\theta_{\mu },G,S|D)\propto P(D|\theta_{\mu },G,S)P(S|\theta_{\mu })P(G|\theta_{\tau })P(\theta_{\tau })P(\theta_{\mu }).\;$$

We can therefore obtain samples from the joint posterior distribution by performing a MCMC method, if we can compute the right-hand side of expression (2.5). The priors *P*(***θ_τ_***) and *P*(***θ_μ_***) are chosen arbitrarily. In order to perform MCMC, we will explore the algorithm to compute the three remaining factors *P*(***D***|***θ_μ_***, ***G***, *S*), *P*(*S*|***θ_μ_***) and *P*(***G***|***θ_τ_***), which appear in the right-hand side of expression (2.5).

As for *P*(*S*|***θ_μ_***), if we have prior knowledge as to the state of the common ancestor, we can simply give the prior probability *P*(*S*). Also, if the mutation process is a non-absorbing Markov process which had lasted for a sufficiently long time before the MRCA, *P*(*S*|***θ_μ_***) is equivalent to the stationary distribution of the process, which is given by the dominant left eigenvector of matrix ***Q***.

Secondly, *P*(***G***|***θ_τ_***) is computed as follows. Let *c_ij_* (1 ≤ *i*, *j* ≤ *n*) denote the number of *j*'s which appear in the *i*-th column of ***G***. In words, *c_ij_* represents the number of times *P_i_* copied the state from *P_j_* in the past *τ* generations. We have

2.6a $$P(G|\theta_{\tau })=\overset{n}{\underset{i=1}{\prod }}\overset{n}{\underset{\;j=1}{\prod }}a_{ij}^{c_{ij}}$$

and

2.6b $$\;\log P(G|\theta_{\tau })=\overset{n}{\underset{i=1}{\sum }}\overset{n}{\underset{\;j=1}{\sum }}c_{ij}\log a_{ij}.$$

Note again that transmission rate *a_ij_* is a function of transmission parameters ***θ_τ_***.

**Figure 5.**
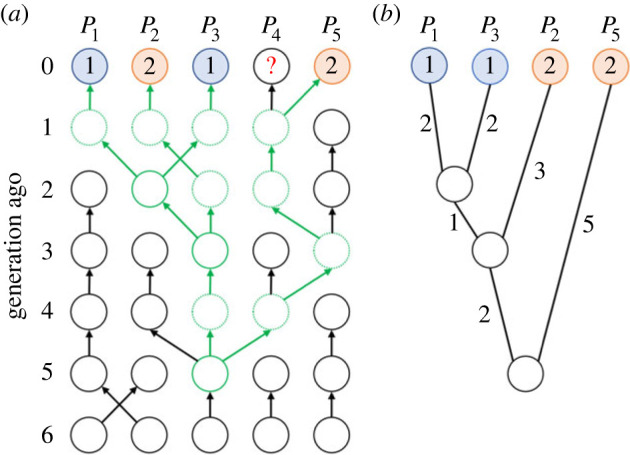
(*a*) Coalescent process based on the past transmission described in figure 3. Green arrows and circles show lineages of the four observed state copies. Green circles represent populations in which a coalescent event happened. (*b*) Genealogical tree generated by the coalescent process. Numbers represent branch length.

Finally, to compute the likelihood *P*(***D***|***θ_μ_***, ***G***, *S*), we consider the coalescent process starting from the *m* state copies of the present generation. Based on ***G***, we track the lineages of these *m* state copies by considering from where populations learned the state in the past. When two populations learn the state from the same population, the two lineages will merge and a common ancestor appears, which is called coalescence in population genetics. If the MRCA does not appear through tracking the genealogy by *τ* generations, we have *P*(***D***|***θ_μ_***, ***G***, *S*) = 0 because it contradicts the prior information that the age of the MRCA is smaller than *τ*. If the MRCA does appear by *τ* generations, we obtain a genealogical tree *T*, where the leaves correspond to the *m* populations whose state is known (figure 5). In this case, we have *P*(***D***|***θ_μ_***, ***G***, *S*) = *P*(***Y***|***θ_μ_***, *T*, *S*).

Since mutation is assumed to be a Markov process and happens independently on every branch of the tree *T*, the likelihood *P*(***D***|***θ_μ_***, ***G***, *S*) = *P*(***Y***|***θ_μ_***, *T*, *S*) can be computed through Felsenstein's tree-pruning algorithm [30,31], which works as follows. Let *v*_1_, … ,*v*_2*m*−1_ denote the nodes of *T*, where *v*_1_, … ,*v_m_* are leaves (i.e. tree taxa), *v_m_*_+1_, … ,*v*_2*m*−2_ are internal nodes and *v*_2*m*−1_ is the root node (MRCA). For *m* + 1 ≤ *i* ≤ 2*m* − 1, let *L_i_*(*j*) denote the probability that all the leaf nodes which are descendants of the node *v_i_* take the same state as the data ***Y***, given *v_i_* takes the state *j*. For the sake of notational simplicity, for 1 ≤ *i* ≤ *m*, we assume *L_i_*(*j*) = 1 if *v_i_* takes state *j* in the data ***Y***, and *L_i_*(*j*) = 0 otherwise. Now, letting *c_i_*_1_ and *c_i_*_2_ be the index of the two child nodes of *v_i_* (*m* + 1 ≤ *i* ≤ 2*m* − 1), we have

$$\begin{array}{c} L_{i}(j)=\left(\sum_{\;j^{\prime }=1}^{k}P(v_{c_{i1}}=j^{\prime }|v_{i}=j)L_{c_{i1}}(j^{\prime })\right)\\ \;\times \left(\sum_{\;j^{\prime }=1}^{k}P(v_{c_{i2}}=j^{\prime }|v_{i}=j)L_{c_{i2}}(j^{\prime })\right). \end{array}$$

Felsenstein's tree-pruning algorithm efficiently computes the likelihood *P*(***Y***|***θ_μ_***, *T*, *S*) = *L*_2*m*−1_(*S*) by recursively applying the equation above from the leaves to the root. The transition probabilities in this equation are determined by the branch length of *T* and the Markov matrix ***Q***, the latter of which is in turn dependent on the mutation parameters ***θ_μ_***.

Based on the above-mentioned algorithms, MCMC methods based on expression (2.5) gives samples from the joint posterior distribution *P*(***θ_τ_***, ***θ_μ_***, ***G***, *S*|***D***), from which we can further obtain samples from the posterior distribution of every model parameter: *P*(***θ_τ_***|***D***), *P*(***θ_μ_***|***D***), *P*(***G***|***D***), and *P*(*S*|***D***). In addition, since a sample of matrix ***G*** can be reduced to a sample of *T*, we can subsequently sample from the posterior distribution of *P*(*T*|***D***) and thus infer the genealogical relationship among the present cultural states. Detailed description of the Metropolis–Hastings algorithm used in MCMC is presented in the online electronic supplementary material.

### 2.6. Inference of unknown states

We have so far described the method to infer the posterior distribution of transmission parameters, mutation parameters and the genealogical relationship of the present states. In this subsection, we aim to infer the posterior probability of the present states which are yet to be observed.

Here, we are interested in inferring the states of *l* unobserved populations $P_{x_{1}},\cdots P_{x_{l}}$, where 0 ≤ *l* ≤ *n* − *m*, 1 ≤ *x_i_* ≤ *n* (1 ≤ *i* ≤ *l*), *x_i_* ≠ *x_j_* (*i* ≠ *j*), *y_i_* ≠ *x_j_* (1 ≤ *i* ≤ *m*, 1 ≤ *j* ≤ *l*) hold true. Thus, ***X*** = {*S*(*x*_1_), … , *S*(*x_l_*)} is considered parameters to be estimated. In this case, we need a prior information about the time to the MRCA of all *m* + *l* populations whose states are either known or to be estimated, so *τ*, the number of rows in matrix ***G***, is defined as the maximum possible age of the MRCA which state copies in *m* + *l* populations share.

**Figure 6.**
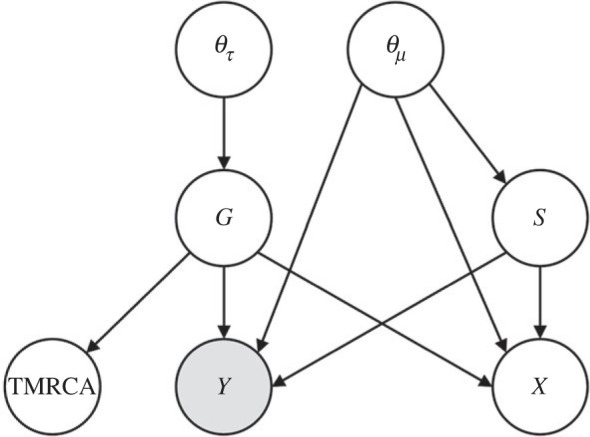
Probabilistic dependency of variables with the set of unknown states denoted by ***X***. Grey circle represents observed variables.

Including ***X***, probabilistic dependency among parameters is depicted as figure 6. Hence, the joint posterior distribution is given by

2.7 $$\begin{array}{cc} P(\theta_{\tau },\;\theta_{\mu },\;G,\;S,\;X|D)\; & =\frac{P(D,\;X|\theta_{\tau },\;\theta_{\mu },\;G,\;S)P(\theta_{\tau },\;\theta_{\mu },\;G,\;S)}{P(D)}\;\\ \; & \;=\frac{P(D,\;X|\theta_{\mu },\;G,\;S)P(S|\theta_{\mu })P(G|\theta_{\tau })P(\theta_{\tau })P(\theta_{\mu })}{P(D)}.\;\; \end{array}$$

Neglecting the marginal likelihood *P*(***D***), we have

2.8 $$P(\theta_{\tau },\theta_{\mu },G,S,X|D)\propto P(D,X|\theta_{\mu },G,S)P(S|\theta_{\mu })P(G|\theta_{\tau })P(\theta_{\tau })P(\theta_{\mu }).\;$$

If we compute the right-hand side of expression (2.8), an MCMC method gives samples from the joint posterior distribution *P*(***θ_τ_***, ***θ_μ_***, ***G***, *S*, ***X***|***D***), which in turn gives inference of the posterior probability of the unknown states *P*(***X***|***D***).

In calculating the right-hand side of (2.8), conditional probabilities *P*(*S*|***θ_μ_***) and *P*(***G***|***θ_τ_***) can be computed in the same way as described in §2.5. To compute the conditional probability *P*(***D***, ***X***|***θ_μ_***, ***G***, *S*), we consider the coalescent process starting from the *m* + *l* populations where the state is either known or to be inferred, obtaining a genealogical tree with *m* + *l* leaves. Letting *T*^′^ denote this tree, the probability *P*(***D***, ***X***|***θ_μ_***, ***G***, *S*) = *P*(***Y***, ***X***|***θ_μ_***, *T*^′^, *S*) can again be computed by the pruning algorithm [30,31].

### 2.7. Heuristic algorithm: Bayesian inference without ***G*** matrix

The method we propose includes a matrix ***G*** with dimension *τ* × *n*, which may make the MCMC method impractically time-consuming as it requires the exploration of a large parameter space, particularly either when the number of populations is large or when the MRCA dates back many generations. For this reason, we will also describe a somewhat heuristic way to avoid the use of the matrix ***G***, whereby, instead of computing the prior probability of ***G***, we draw a sample from the prior distribution of the genealogical tree *T* (or *T*^′^ if *l* > 0) by directly simulating the coalescent process. More specifically, starting from the *m* observed populations $P_{y_{1}},\cdots ,P_{y_{m}}$ (and *l* unobserved populations $P_{x_{1}},\cdots P_{x_{l}}$ if applicable), we simulate the lineages of these cultural states by recursively retracing past populations from which current populations copied the state. Note that, through this ancestral process, *a_ij_* signifies the probability that a lineage belonging to *P_i_* is transferred to *P_j_*. Since every population takes only one state at a time, two lineages coalesce when they belong to the same population. The process is terminated when all *m* + *l* lineages have coalesced, yielding the tree *T*^′^, which contains information of tree topology, branch lengths, and populations to which ancestral nodes belong. Given transmission parameters ***θ_τ_***, the probability of obtaining a tree *T*^′^ through this process is given by *P*(*T*^′^|***θ_τ_***). As well as equation (2.8), we have

2.9 $$P(\theta_{\tau },\theta_{\mu },T^{\prime },S,X|D)\propto P(D,X|\theta_{\mu },T^{\prime },S)P(S|\theta_{\mu })P(T^{\prime }|\theta_{\tau })P(\theta_{\tau })P(\theta_{\mu }),$$

where *P*(***D***, ***X***|***θ_μ_***, *T*^′^, *S*) can again be computed by the pruning algorithm.

We summarize the workflow of the Bayesian inference without the matrix ***G*** as follows:

(A) Sample a set of transmission parameters ***θ_τ_*** from the prior *P*(***θ_τ_***).
(B) Simulate the ancestral (coalescent) process based on ***θ_τ_*** sampled in (A) and generate a tree *T*^′^.
(C) Repeat (A) and (B) for enough iterations and obtain multiple trees regarded as a sample from the prior distribution *P*(*T*^′^, ***θ_τ_***) = *P*(*T*^′^|***θ_τ_***)*P*(***θ_τ_***).
(D) Perform MCMC and obtain the joint posterior distribution *P*(***θ_τ_***, ***θ_μ_***, *T*^′^, *S*, ***X***|***D***) using equation (2.9). We approximate the prior *P*(*T*^′^, ***θ_τ_***) = *P*(*T*^′^|***θ_τ_***)*P*(***θ_τ_***) by the empirical distribution obtained in (C).
(E) Obtain the posterior distribution of each parameter: *P*(*T*^′^|***D***), *P*(***X***|***D***), *P*(*S*|***D***), *P*(***θ_τ_***|***D***), and *P*(***θ_μ_***|***D***).

The core of this method consists in the approximation of the probability *P*(*T*^′^|***θ_τ_***) by the prior trees sampled through the Monte Carlo simulation of the coalescent process (B). In the electronic supplementary material, we test the performance of both the algorithm with ***G*** and the heuristic algorithm and show that the latter is remarkably more efficient in terms of the effective sample size and run time (electronic supplementary material, table S1, S2).

## 3. Example on a square grid

### 3.1. Model assumptions

In this section, we provide an example to which our method is applied. We consider an *L* × *L* square grid where each square represents a population (*n* = *L*^2^). Regarding each square as a node of the network, an edge is assigned between every pair of squares which share a side (i.e. each population can copy the state from four surrounding populations). Transmission rate is assigned by

3.1 $$a_{ij}=\{\begin{array}{c} 1-d\times \deg(P_{i})\;\;(if\;i=j)\\ d\;\;(if\;P_{i}\;\mathrm{is}\;\mathrm{adjacent}\;\mathrm{to}\;P_{j})\\ 0\;\;(\mathrm{otherwise}) \end{array}.\;$$

Hence, the transmission parameter of this model is ***θ_τ_*** = {*d*}. We consider a two-state model (*k* = 2) where the mutation rate is given by

$$Q=\left(\begin{array}{cc} 1-q_{12} & q_{12}\\ q_{21} & 1-q_{21} \end{array}\right).$$

Thus, two mutation rates *q*_12_ and *q*_21_ constitute the mutation parameters ***θ_μ_*** of the model. The power of this matrix can be calculated analytically as follows:

3.2 $$Q^{t}=\left(\begin{array}{cc} \pi_{2}\lambda^{t}+\pi_{1} & -\pi_{2}\lambda^{t}+\pi_{2}\\ -\pi_{1}\lambda^{t}+\pi_{1} & \pi_{1}\lambda^{t}+\pi_{2} \end{array}\right),$$

where

3.3a $$\;\lambda =1-q_{12}-q_{21},$$

3.3b $$\;\pi_{1}=\frac{q_{21}}{q_{12}+q_{21}},$$

3.3c $$\mathrm{and}\;\pi_{2}=\frac{q_{12}}{q_{12}+q_{21}}.\;$$

We will use equation (3.2) in computing the likelihood via Felsenstein's pruning [30,31]. In addition, the prior of the ancestral state *P*(*S*|***θ_μ_***) is given as the stationary distribution of the Markov process, so we have *P*(*S* = 1|***θ_μ_***) = *π*_1_ and *P*(*S* = 2|***θ_μ_***) = *π*_2_.

### 3.2. Inference of the mutation parameters and unobserved states

**Figure 7.**
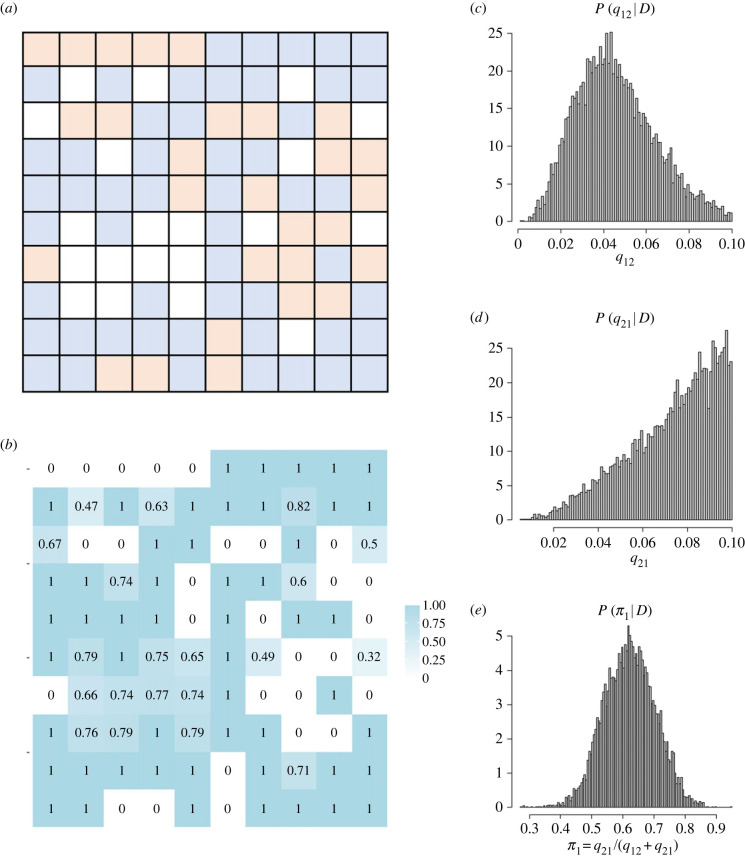
Simulation on a two-dimensional square lattice with 10 populations arranged each side. (*a*) Observed states of the populations (squares) used for the Bayesian inference. Blue and orange represent states 1 and 2, respectively, and white represents the absence of data (i.e. unobserved population). (*b*) Posterior probability of each population having the state 1. (*c*) Posterior probability distribution of *q*_12_. (*d*) Posterior probability distribution of *q*_21_. (*e*) Posterior probability distribution of *π*_1_.

Figure 7*a* shows an example of the observed spatial pattern of cultural states (*L* = 10, *n* = 100), in which states in 80 populations are known and the rest are to be inferred by the model (*m* = 80, *l* = 20). The state data in figure 7*a* were created by simulating the transmission and mutation events over 5 × 10^5^ generations as described in §3.1 with parameter values *d* = 0.15, *q*_12_ = 0.04, *q*_21_ = 0.08 and by concealing states of *l* = 20 populations to represent unobserved states.

We applied the algorithm with matrix ***G*** described in §§2.5 and 2.6 to this case. In the implementation of Bayesian inference from the state data, the prior distributions for the parameters *q*_12_ and *q*_21_ were *U*(0, 0.1), where *U*(*x*, *y*) represents the uniform distribution with the minimum and maximum values being *x* and *y*, respectively. Unfortunately, when *n* is large, the parameter *d* does not converge to the stationary distribution within a practical computation time (see electronic supplementary material). We thus confined our analysis by setting *d* as a constant value 0.15, meaning that we are assumed to already know the true value of this parameter.

After obtaining the joint posterior distribution, we visualized the posterior probability of each population being in state 1 (figure 7*b*), the posterior distributions of the mutation rates (figure 7*c,d*), and the stationary probability of state 1 (figure 7*e*) given by equation (3.3*b*).

Figure 7*b* shows that populations are likely to take the same state as the populations nearby, which is not surprising because our model assumes a network structure of populations in which cultural states transmit only between neighbouring populations. The result also shows a tendency that populations have a higher posterior probability of state 1 than the posterior probability of state 2.

Overall, although our algorithm with matrix ***G*** can estimate the posterior distributions of mutation rates and unknown states, the posterior distributions of mutation rates seem relatively broad, indicating that we cannot infer the parameter values with a high credibility. This is also the case for the unknown states; posterior probabilities of being in state 1 range from 0.32 to 0.79, meaning that the dataset only enables us to specify the state with the credibility of up to 80%. We tested the accuracy of the inference of unobserved states in the electronic supplementary material.

### 3.3. Reconstruction of the genealogical tree and inference of the transmission parameter

**Figure 8.**
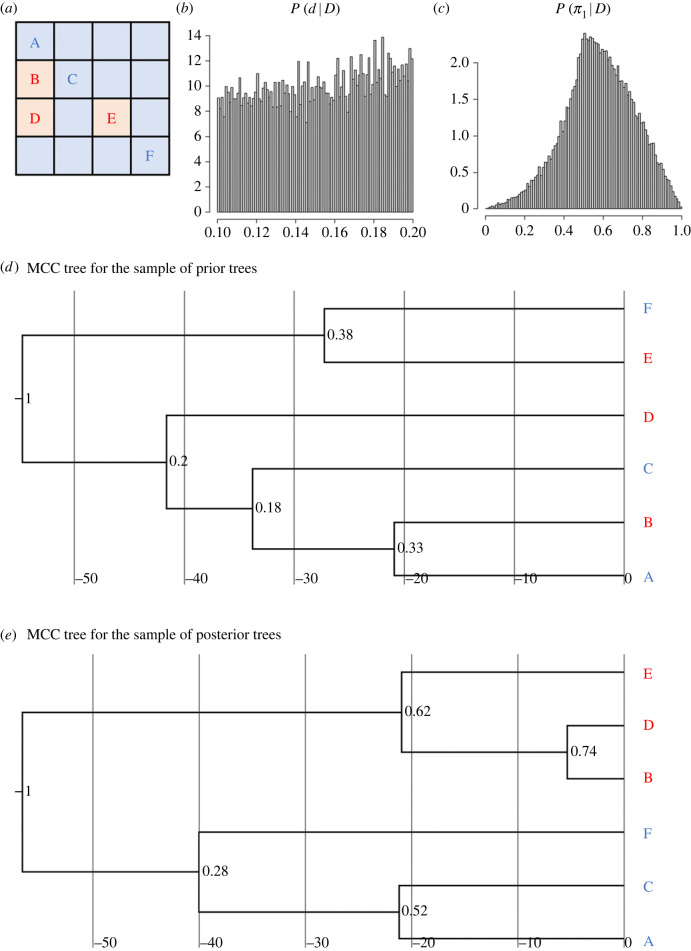
Simulation on a two-dimensional square lattice with four populations arranged each side, where the genealogical relationship among multiple state copies observed is inferred. (*a*) Observed states of the populations (squares) used for the Bayesian inference. Blue and orange represent states 1 and 2, respectively. States labelled A to F are the taxa of the genealogical tree. (*b*) Posterior distribution of *d*. (*c*) Posterior distribution of *π*_1_. (*d*) The maximum clade credibility (MCC) tree derived from the sample of prior trees, where the colours of the taxa label represent cultural states (i.e. blue and red correspond to states 1 and 2, respectively). Values assigned to the root and internal nodes represent clade probabilities. (*e*) The maximum clade credibility (MCC) tree derived from the sample of posterior trees.

If the number of populations is small, we can both infer the transmission parameters and reconstruct the genealogical tree from the spatial distribution of the state. We conducted the simulation with the state data with *L* = 4, *n* = 16, *m* = 16, whereby the genealogical relationship among six state copies is inferred (figure 8*a*). This state data was created by simulating cultural transmission over 10^4^ generations with parameter values *d* = 0.15, *q*_12_ = 0.01, *q*_21_ = 0.02. By contrast with the previous subsection, the heuristic algorithm was employed to perform Bayesian inference, in which 5 × 10^6^ prior trees were sampled via the coalescent simulation. We used the prior distribution *U*(0.1, 0.2) for *d* and *U*(0, 0.03) for both mutation rates *q*_12_ and *q*_21_. To summarize the prior *P*(*T*) and posterior *P*(*T*|*D*) of genealogical trees, we produced the maximum clade credibility (MCC) trees from both prior and posterior samples (figure 8*d,e*). We used the TreeAnnotator program distributed with BEAST2 [32] to build the MCC tree from the sampled trees, and the resulting MCC tree was visualized by the browser-based phylogenetic viewer IcyTree available at https://icytree.org/ [33].

Comparison between figure 8*d* and *e* indicates that, whereas state copies observed in nearby populations tend to form clades in the prior trees, populations with the same state are more likely to form clades in the posterior trees. The result also suggests that the time to the MRCA (i.e. length from the tree root to the leaves) is on average longer, as slightly as it is, in posterior trees than in prior trees. It is also notable that two populations with the same state tends to have a young common ancestor in the posterior tree.

These results are quite intuitive and straightforward because the prior probability $P(T)=\int P(T|\theta_{\tau })P(\theta_{\tau })d\theta_{\tau }$ does not include information about the spatial pattern of cultural states, so the prior trees are formed merely in view of the locations (nodes) where the states are observed. Since the prior distribution of mutation rates ranges within small values, there must be a relatively long branch for mutations to happen, which explains the longer time to the MRCA in posterior trees. However, since our model considers the variation of only one cultural trait, the posterior values of clade probabilities tend to be moderate, unlike purpose-built models for phylogenetic reconstruction which accommodate variation in tens or hundreds of traits.

## 4. Discussion

In this article, we introduced a Bayesian framework to estimate parameters which determine the transmission and mutation rates, and cultural states yet to be observed. As the Bayesian inference is conducted with the aid of a phylogenetic approach, we may also infer the genealogical history of the copies of cultural states that we observe in the present generation. In addition to the exact MCMC relying on equations (2.5) and (2.8), we have also developed a heuristic algorithm to approximate the posterior distributions by sampling from the prior distribution of phylogenetic trees through the simulation of the coalescent process. We have also presented numerical examples in which our methods were applied to estimating the posterior distribution based on a two-dimensional square grid.

We first summarize the pros and cons of our method based on the results of numerical analysis. A major advantage of this model is that it helps the inference of model parameters concerning transmission and mutation from the spatial data. It is also of interest to infer the states of unobserved populations from the states of nearby populations, as the number of survey locations or populations covered by field work is limited. By contrast, the limitation of the method is that the posterior distributions are relatively broad (figure 7) when we use an uninformative prior like uniform distribution, so that the inference of model parameters with a good precision is impossible. This is not a problem of Bayesian inference but the characteristic of the model set-up itself (see §§2.1 and 2.2). We suggest that a strong prior, based on assumptions or prior knowledge, be used in applying the model to empirical data to make inference for a practical purpose. Another limitation is that MCMC in our methods does not always converge to the posterior distribution within a practical computation time: the heuristic method does not work well when the number of observed populations is large, whereas MCMC with ***G*** does not enable transmission parameters to converge (see electronic supplementary material).

To compare the current study with past studies, we discuss a series of research in a growing field called phylogeography, which treats biological or cultural evolution through phylogenetic approaches with geographical information. These methods reconstruct the phylogenetic tree while simultaneously inferring the unobserved locations of ancestors (internal nodes of the tree), to give inference to the diffusion rate of lineages. Some previous models represented space as continuous states and regarded diffusion as the random walk [34], which was further extended to take into consideration the difference in the rate of diffusion among tree branches (relaxed random walk, RRW) [35]. On the other hand, like the model of the current article, another previous study considered space as discrete states [36], and the location which each internal node occupies is also a parameter of Bayesian inference. These methods have been employed to trace the spatial patterns of pathogen dispersal [35,36] and to infer the originating place of the Indo-European language family [26].

The major difference between our model and those previous models is that our model does not allow the coexistence of different cultural states within a single population and treats the coalescent process of lineages more rigorously at the cost of computation time. Standard methods in phylogenetic reconstruction with spatial diffusion only consider the state of tree nodes in the calculation of tree likelihood, so two lineages may occupy the same location at the same time. On the other hand, our method, either the model with matrix ***G*** or the heuristic algorithm which samples prior trees via Monte Carlo simulation, is a time-discrete model and considers the location of each lineage every generation. Our model can thus rigorously trace the coalescent process of known state copies under the premise that each location can only take one cultural state, which is particularly essential when the number of populations (network nodes) and the number of observed populations are of the same order of magnitude. Nevertheless, it is also possible to model the coexistence of two states by introducing the third state which corresponds to the mixture of the two states.

We consider the application of this method to empirical data. One possible field for application is dialectology, because the horizontal transmission exerts significant influence on the dialect-level variation of language. Despite little attention to tree thinking paid by dialectologists, our method with the single-trait tree seems appropriate for dialectology known for the slogan ‘every word has its own history'. Regarding empirical data, geographical distributions of lexical, phonological, morphological and syntactical variants are recorded in the form of a map called linguistic atlas (e.g. Linguistic Atlas of Japan [37]). Since a linguistic atlas is composed of multiple different maps corresponding to different surveyed items, it would be intriguing to apply our single-trait model independently to each map, to elucidate what kind of linguistic features are likely to emerge or disappear. In applying the model to a linguistic atlas, the populations in our model would probably represent either distinct cities or lattice sites assigned at a regular interval. Our one-population-one-state model seems to fit the nature of the dialect; Individuals do not independently adopt the speech variant but they tend to conform to the local majority, homogenizing the variant within each population.

Moreover, theoretical studies in linguistic geography have represented the transmission of linguistic traits as a function of geographical distance and population size of each location, such as the Gaussian interaction kernel [29], gravity model [38] and long-distance communication between cities [39], which would help us to define the matrix ***A*** = (*a_ij_*) of our Bayesian framework. In applying the model to dialectology, we can use the mutation models of previous studies as the ***Q*** matrix of our framework. For example, if we model the presence/absence of a lexical item, which never reappears once it is lost, we may use the pseudo-Dollo model [40], with the three states representing ‘initial', ‘present’ and ‘removed'. If we wish to model the competition of two words, the prior of the mutation rate between the two states should be confined within an extremely low value, such as *U*(0, 10^−10^), which would model the assumption that the same word emerges only once in history.

Archaeology and ethnology are also promising fields for application, since a large body of empirical data from fieldwork has been created with spatial information. Variation in archaeological and ethnological traits over different populations or survey locations was used to detect the horizontal transmission by previous work of cultural evolution [41]. Our model may also be suitable to study the spatial pattern of cumulative cultures [42], if appropriate data are available. In this case, cultural states may represent culture levels (e.g. presence or complexity of skills) and the mutation parameters would be the rates at which the culture level increases and decreases. However, it must be noted that our model assumes neutral evolution of trait variants, that is, the trait is copied with the same probability regardless of its state. This might not be realistic in the study of cumulative culture, which has been modelled with a biased learning strategy toward higher skill levels [42,43].

Finally, we discuss a further extension of our model. A suggestion for future research is to integrate the population-dependent variation of mutation rates into the model to infer which populations are innovative and which are conservative. This extension can take advantage of our model feature whereby coalescent process is simulated generation by generation; we can assign a greater branch length when the lineage passes a population prone to mutation. Another way of extension is to reduce the computation time of the MCMC method by reducing the dimension of ***G***, especially when the mutation of cultural states is frequent. Instead of tracing the past transmission by the maximally possible age of MRCA (i.e. *τ*), we may abort the coalescent process at some generation *τ*′ ( < *τ*), where *τ*′ is larger by an order of magnitude than the reciprocal of mutation rates. If we have *i* lineages when we abort the coalescent process at generation *τ*′, we have *i* genealogical trees whose taxa are a subset of the *m* + *l* populations. The likelihood can be approximately computed by independently applying the pruning algorithm to each tree, which enables us to reduce the dimension of ***G*** from *τ* × *n* to *τ*′ × *n*. However, the inference of the genealogy is impossible in this way.

## Data Availability

The data and code for this article are available from the Zenodo repository: https://doi.org/10.5281/zenodo.7315710 [44]. Supplementary material is available online [45].
