# Corrigendum to: ‘Spatial evolution of human cultures inferred through Bayesian phylogenetic analysis’ (2023) by Takahashi and Ihara

**DOI:** 10.1098/rsif.2023.0249

**Published:** 2023-05-17

**Authors:** Takuya Takahashi, Yasuo Ihara

*J. R. Soc. Interface* **20**, 20220543. (Published online 4 January 2023) (https://doi.org/10.1098/rsif.2022.0543)

The authors regret that they have found two errors in the article.

1. In figure 2 of the original article, the cultural state of *P*_2_ at generation *t* + 1 abruptly changes from 2 to 1 without a red star representing the mutation event, which does not accurately illustrate the model assumptions. Here, we present the correct version of the figure.

2. In §3.2, paragraph 5, the first sentence ‘Overall, although our heuristic algorithm can estimate…’ should read ‘Overall, although our algorithm with matrix ***G*** can estimate…’.

The errors above do not affect the validity of the original article.

**Figure 2.**
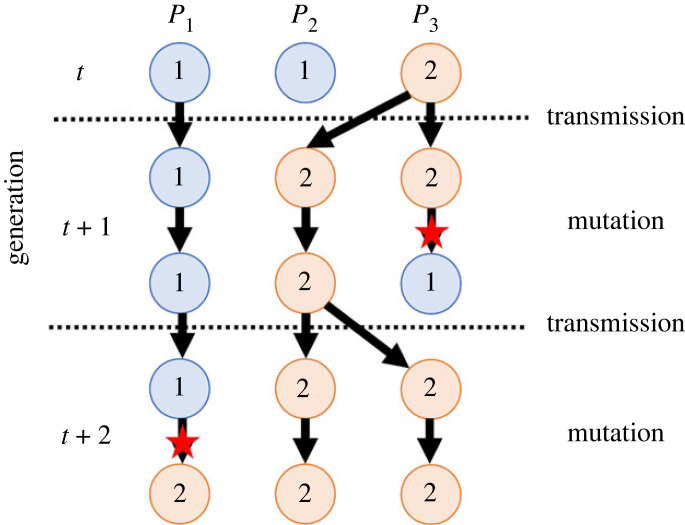
Dynamics of cultural states of three populations in three consecutive generations (*n* = 3, *k* = 2): (circles) populations, (blue) state 1, (orange) state 2, (arrows) inheritance of cultural states and (red stars) mutation events.

This has been corrected on the publisher's website.

